# A Conversation with Alba Álvarez-Martín

**DOI:** 10.1021/acscentsci.4c01408

**Published:** 2024-09-10

**Authors:** Jonathan Feakins

Growing up in Salamanca, Spain, Alba Álvarez-Martín saw art taking shape around her—literally. Her father was a sculptor, but she didn’t develop the same artistic skill. Instead, her passions leaned toward physics, math, and chemistry. Today, she’s found her calling as a cultural heritage scientist at Amsterdam’s Rijksmuseum, devising techniques to analyze aging works of art.

**Figure:**
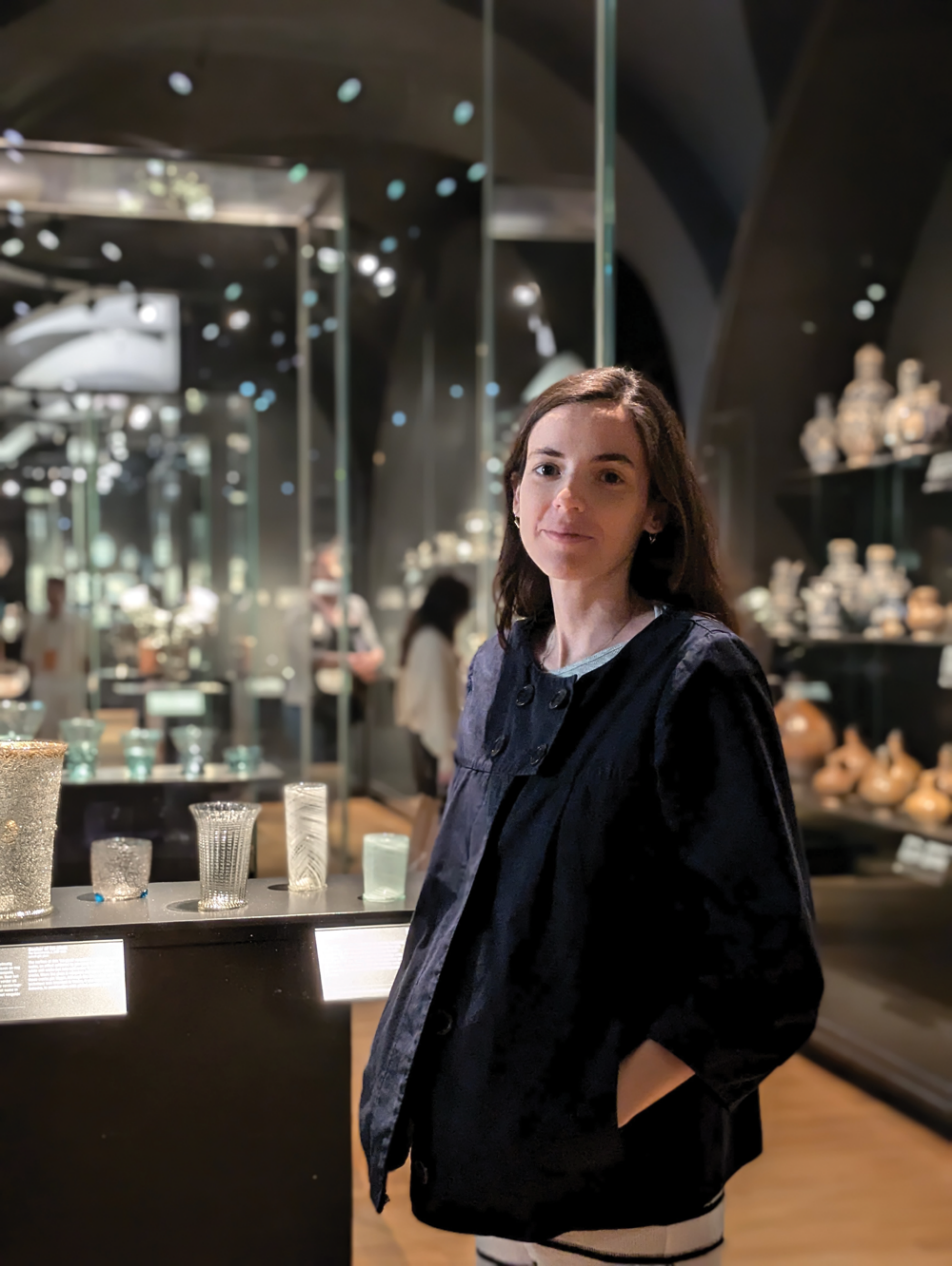
Alba Álvarez-Martín stands amid the special collections at the Rijksmuseum. Credit: Frederik Vanmeert.

“I cannot make it,” Álvarez-Martín admits. “But I can conserve it!”

Her drive inspired her to borrow a technique from medical imaging and deploy it in her art conservation work. Last year, her team reported using matrix-assisted laser desorption ionization mass spectrometry imaging (MALDI-MSI) to precisely map out where compounds occur in the thin cross section of a painting, a method that could be used to glean molecular details about each layer of paint that artists or previous conservators have laid down (*Anal. Chem.* 2023, DOI: 10.1021/acs.analchem.3c03992).

This technique may provide invaluable insight into some of the gripping questions in cultural heritage: how artists make their art, how the colors have changed over time, and what can be done to prevent degradation. Jonathan Feakins spoke with Álvarez-Martín about finding inspiration from medicine, recreating centuries-old pigments, and pursuing her lifelong obsessions. This interview was edited for length and clarity.

## How does analysis of art, like the analysis you’re doing with MALDI-MSI, inform the conservation process?

Organic pigments often contain a chromophore, a molecule with alternating single and double bonds responsible for the color. However, the double bonds can be very sensitive to light, and that light can catalyze the breakdown of these conjugated systems—[resulting in] the loss of color. We can propose the right light conditions to make the pigment fade slower, and the museum can set different policies regarding their lighting system.

But this type of analysis can also give a lot of information from the art historical point of view—for example, how the painting was created by the artist and how the composition has evolved over the centuries. Sometimes we cannot identify the original pigments, but we detect degradation products. This can inform us about the original material that the artist used. We can try to backtrack to the original composition, like reverse engineering.

## Upon publication of your paper, you said that MALDI-MSI allowed you to “turn a dream (and an obsession) into a reality.” What did you mean by that?

My obsession has always been, how can I use mass spectrometry in a less invasive manner, in a way that keeps the spatial distribution of the pigments in the sample? Second, how can we make the results more visual to produce images that are easy for people outside the research field to interpret?

The main advance is being able to visualize where the degradation products are in a cross section of a painting and where the original pigments are. With traditional techniques, we had to extract the analytes from the bulk sample, and during this process we lost the spatial information. Basically, we knew which fragments were present, but we didn’t know where they were.

## Can you explain, broadly, how MALDI-MSI works?

MALDI-MSI is a tool that can visualize the distribution of molecules without extraction, purification, and separation of the sample’s components. After collecting a mass spectrum at one spot, the sample is moved to reach another region, and so on, until the entire sample is scanned. In the resulting spectra, masses that correspond to the lake pigments and their degradation products can be made to map where they appear in a multilayered sample.

MALDI-MS imaging is widely applied in the biomedical field to image peptides, proteins, lipids, and carbohydrates. For example, if doctors are analyzing a sample tissue, they can distinguish between cancerous tissue and normal tissue based on the distribution of these biomolecules. So this is really useful for a thin section of tissue.

My idea was: if I have a thin cross section of a painting, maybe I can see the distribution of the original pigment and its degradation products.

## For the study, you created your own in-house batch of geranium lake, a bright-pink pigment often found in the works of Van Gogh. What have you done with MALDI-MSI so far, and how will you approach the paintings themselves?

First, it is really important to make sure that the method that we are optimizing works with mock-up samples before analyzing real samples. In our case, we prepare paint samples in the lab following historical recipes: we synthesized the lake pigment by precipitating the dye with a metallic salt, we prepared the oil paints by mixing that lake pigment with linseed oil, and we exposed them to artificial light to age them.

We investigated the efficacy of MALDI-MS imaging in oil paint samples containing a mixture of two organic pigments—geranium lake and lead white, a mixture often employed in Van Gogh’s oeuvre. The analysis provided valuable molecular information on the degradation pathways of geranium lake in specific paint layers.

In a parallel project at the Rijksmuseum, we are using more advanced instrumentation in collaboration with the group of Professor Ron Heeren (at Maastricht University) to investigate the red lakes used by Rembrandt in The Night Watch. We are in the process of tuning and adapting the instrumentation before analyzing real samples coming from the painting.

## Where is there room for future developments in using MALDI-MSI for cultural heritage?

I think that both the imaging and portable instrumentation are approaches that I would like to see in the museum community in the next decade—and I see the field moving in that direction! Not only being able to map those degraded products but also to do portable MS in the museum so that we don’t need to take a sample from a piece of art and move it to the lab to perform destructive analysis. We could actually move the instrument into the museum.

## What is your next dream or obsession?

I think that making mass spectrometry more accessible in museums is going to be my obsession for life.

*Jonathan Feakins is a freelance contributor to*Chemical & Engineering News*, an independent news publication of the American Chemical Society. Center Stage interviews are edited for length and clarity.*

